# *Eimeria* proteins: order amidst disorder

**DOI:** 10.1186/s13071-022-05159-0

**Published:** 2022-01-24

**Authors:** Joshua Seun Olajide, Zigang Qu, Shunli Yang, Oyeseyi Joshua Oyelade, Jianping Cai

**Affiliations:** 1 State Key Laboratory of Veterinary Etiological Biology, Key Laboratory of Veterinary Parasitology of Gansu, Lanzhou Veterinary Research Institute, Chinese Academy of Agricultural Sciences, Lanzhou, 730046 China; 2 Jiangsu Co-Innovation Center for Prevention and Control of Animal Infectious Disease and Zoonoses, Yangzhou, 225009 China; 3 Centre for Distance Learning, Obafemi Awolowo University, Ile-Ife, Nigeria; 4 Natural History Museum, Obafemi Awolowo University, Ile-Ife, Nigeria

**Keywords:** Apicomplexa, Coccidiosis, *Eimeria*, Parasite, Protein, Secretion, Antigen

## Abstract

Apicomplexans are important pathogens that cause severe infections in humans and animals. The biology and pathogeneses of these parasites have shown that proteins are intrinsically modulated during developmental transitions, physiological processes and disease progression. Also, proteins are integral components of parasite structural elements and organelles. Among apicomplexan parasites, *Eimeria* species are an important disease aetiology for economically important animals wherein identification and characterisation of proteins have been long-winded. Nonetheless, this review seeks to give a comprehensive overview of constitutively expressed *Eimeria* proteins. These molecules are discussed across developmental stages, organelles and sub-cellular components vis-à-vis their biological functions. In addition, hindsight and suggestions are offered with intention to summarise the existing trend of eimerian protein characterisation and to provide a baseline for future studies.

**Graphical Abstract:**

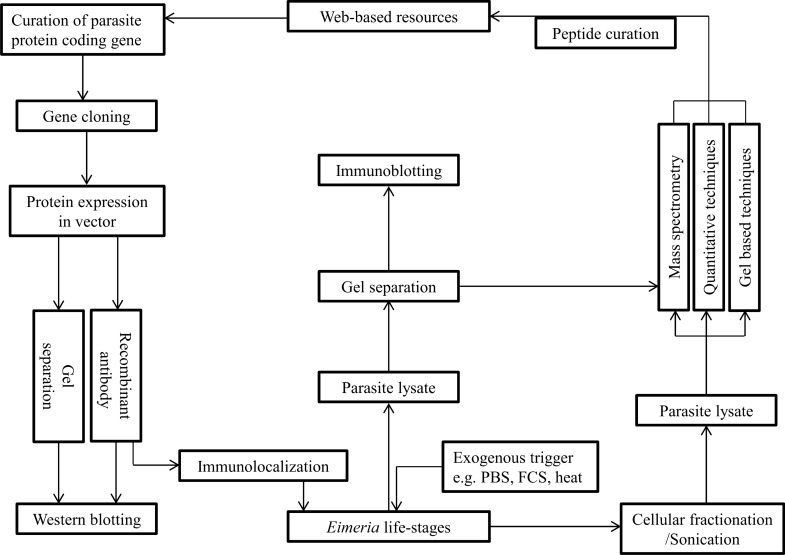

## Background

*Eimeria* is the largest genus in the phylum Apicomplexa with > 1800 described species [1, 2] and one of the most speciose eukaryotic taxa [3, 4]. Eimerians share some similarities with coccidian genera such as *Cyclospora*, *Cystisospora, Sarcocystis*, *Toxoplasma, Neospora*, *Epieimeria*, *Karyolysus* and *Hammondia* but are less related to *Cryptosporidium* and remotely to *Plasmodium, Theileria* and *Babesia* [5, 6]. *Eimeria* are obligate intracellular parasites in all classes of vertebrate [7] with absolute host and tissue specificity [8, 9]. *Eimeria* cause coccidiosis, the most important parasitic disease in poultry [10–14], which can transfer easily among congeneric hosts [15, 16].

Aside from oocyst morphology, *Eimeria* species are classified by mitochondrial cytochrome c oxidase subunit I, 18S ribosomal DNA and RNA, internally transcribed spacer [13, 15, 17, 18] and mitochondrial *cox1*, *cox3* and *cytb* [19]. For avian *Eimeria*, mitochondrial and whole-genome phylogeny could be defining [20]. Species of turkey are polyphyletic [21, 22]. Bovine *E. bovis* and *E. zuernii* and rabbit-infecting *E. stiedai* and *E. flavescens* are cladistic [21, 22]. Besides, with plastid ORF470, *E. falciformis* and *E. nieschulzi* are more related [15]. However, mitochondrial, whole-genome [23], single-oocyst isolation and comparable genetic studies would differentiate many species. Pathologically, haemorrhage and malabsorption are common in *Eimeria*-infected chickens [24] whereas *E. falciformis* cause murine catarrhal enteritis [25] and *E. nieschulzi* induces diarrhoea in rats [26]. *Eimeria bovis* and *E. zuernii* cause petechial haemorrhage and catarrhal enteritis respectively [21, 27] while cholangitis and diarrhoea symptoms of *E. stiedai* infection in rabbits [22]. Other eimerian pathologies are described in [28–31].

**Fig. 1:**
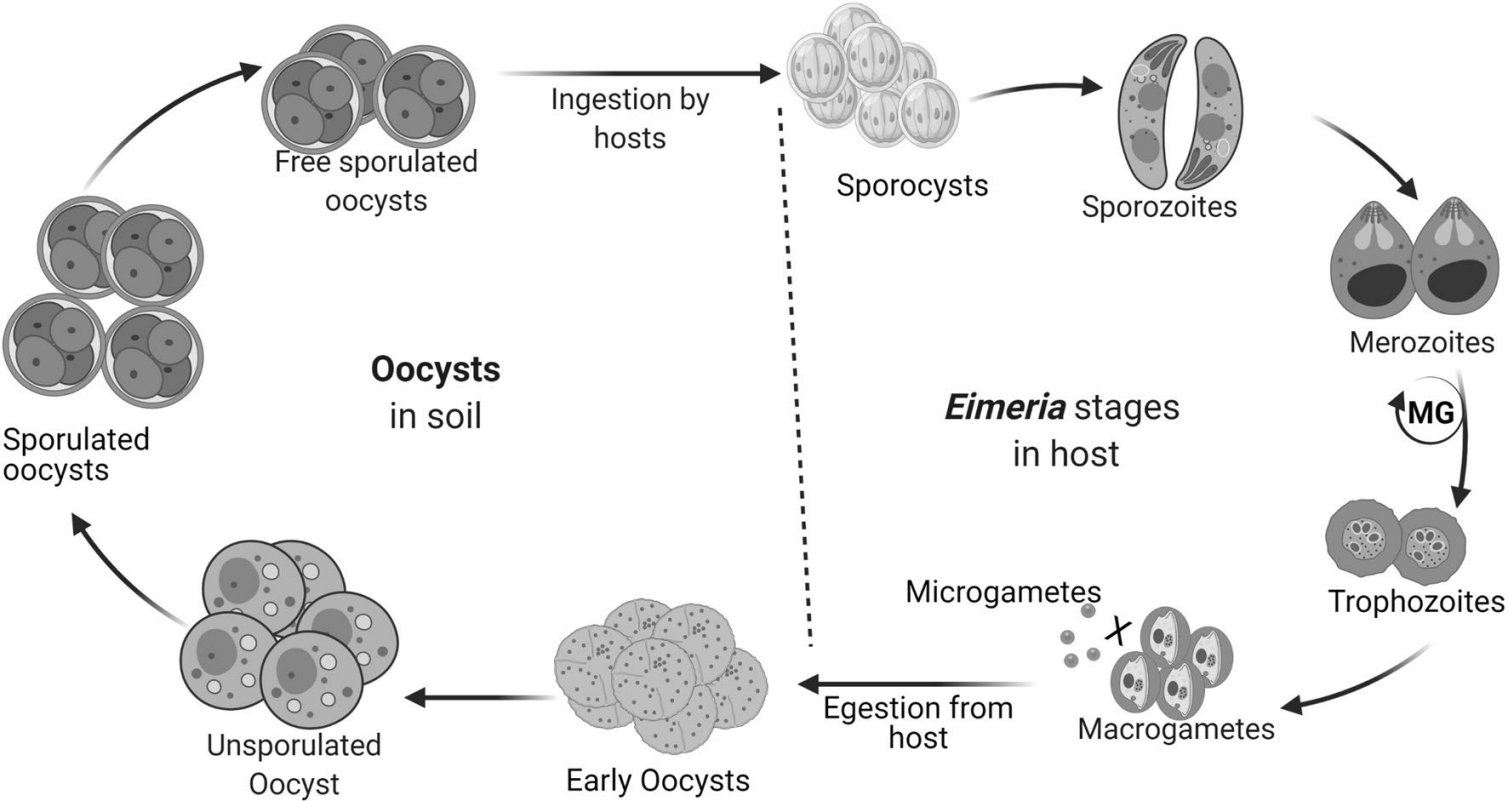
Major developmental stages of *Eimeria*. *Eimeria* life-stages within the host occur once except in merozoite where there can be two or more generations. Only sporulated oocysts are infective and may remain inactive until excystation is activated by enzymatic reaction in the host gut to liberate two to four sporocysts from which sporozoites are released. The sporozoites then transform into merozoites, trophozoites, gametocytes and then oocysts, which are released with host egesta. The distinction between early and late oocysts cannot only be explained away by sporulation as oocysts may remain unsporulted for a long time in the environment. MG: merozoite generation

Eimerians life stages comprise schizogony (asexual) and gametogony (sexual) in the host while sporogony (asexual) occurs outside the host [32, 33]. Susceptible hosts become infected after ingestion of sporulated oocysts containing two to four sporocysts. From each sporocyst, two motile sporozoites are liberated to invade host intestinal epithelium and form non-motile trophozoites. Intracellular sporozoites later transform into spheroidal schizonts and continue asexual development or further nuclear division to form merozoites by merogony. Merozoites released from schizonts can re-invade new epithelial cells or develop into micro- and macro-gametes, which eventually fuse to form zygote and oocyst [34]. However, the number of merozoite generations (MGs) varies with species [6] and the entire life cycle (Fig. 1) depends on gene expressions [34, 35].

*Eimeria* sporozoites, merozoites and trophozoites (zoite stages) possess sub-cellular structures [36] such as apicoplasts, rhoptries, micronemes, conoids, dense granules, polar rings and sub-pellicular microtubules [37] as well as Golgi apparatus, cytoskeleton-associated structures [38–40], inner membrane complexes and acidocalcisomes [32, 41] and, specifically, refractile bodies (RBs) and amylopectin granules [32, 42]. Apical, membrane-bound and heat shock proteins and proteases have been well studied [43]. Succinctly, this review focuses on chicken-infecting *Eimeria* proteins and a few other species of cattle, buffaloes, rabbits, mice and rats. Published works were searched in popular databases for *Eimeria* secreted and recombinant proteins vis-à-vis their functions with a view to presenting a conspectus on *Eimeria* proteins. After brief remarks on protein-coding genes, functions of *Eimeria* proteins across developmental stages and organelles are discussed compared with other apicomplexans. Hindsight and insights are offered for future studies.

## A glimpse into protein gene profiles

*Eimeria* with known genomic sequences have nuclear genomes that enclose 42 to 72 Mbp DNA scattered in 14 chromosomes that range between 1 and > 7 Mb. In addition, mitochondrial (~ 6200 bp) and apicoplast (~ 35 kb) genomes as well as double-stranded RNA segments have been described in many species [2, 8, 44, 45]. Generally, eimerian genomes have segmented chromosomal structure with tri-nucleotide (CAG) repeats in the protein-coding region [44] that predominantly transcribe homopolymeric amino acid repeats [46, 47]. At genomic level, protein coding sequence repeats are well conserved among *Eimeria* but the frequency and location vary among species and strains [44]. Whole-genome gene identification has shown that eimerians have between 5000 to > 10,000 predicted protein coding genes [2, 8]. Meanwhile, stage-specific transcription patterns are estimated to comprise around 4000–5500 genes [48] in which *Eimeria* with a complete genome sequence could express 6000 to 9000 proteins across all developmental stages [33](Fig. 1). Essentially, chicken-infecting species have a significant number of protein-coding genes and larger gene sizes than *T. gondii*, *P. falciparum*, *T. annulata* and *C. parvum* [46, 47].

Oocyst wall protein genes—*owp6* and *howp1* from *E. tenella* oocysts and gametocytes [49], *owp6* and *owp2* in *E. nieschulzi* sporulated oocysts [26] and putative *E. falciformis owp13* and *E. nieschulzi owp13* [50]—have been mapped. Prominent genes in avian and rodent *Eimeria* oocyst development are homologous *gam56* and *gam82* [51] but unlike *gam82*, *gam56* can undergo alternative splicing in *E. nieschulzi* [52]. In addition, *E. tenella gam22*, *gam230* and *gam59* [49] and *E. necatrix gam22* have been annotated [53]. *E. tenella* and *E. necatrix* have 28 rhoptry kinase genes, *rops*, which showed divergence in *E. acevulina* and *E. maxima* [46, 54]. Putative *rop21*, *rop23*, *rop30* and *rop35* and putative dense granule protein genes (*dgs*), *dg10*, *dg11* and *dg32* have been reported in *E. necatrix* [55]. Also, some rhoptry neck protein genes (*ron*s) are expressed by more than one gene in *E. tenella* [56] and *E. necatrix* [55]. Nevertheless, many *dg*s in *T. gondii* are absent in *E. falciformis* genome [2]. Microneme protein genes (*mic*s) that have been predicted and mapped include *mic1-5, 7–9, ama-1*, mic*13* and other four *mic* orthologues [55, 57]. In all, *mic2* has been found highly conserved among *E. tenella* strains [58]. Although *mic1-5*s occupy different chromosomal loci, *mic*s transcriptional and translational regulations are sufficiently synchronous with oocyst sporulation [39]. Yet, unsporulated oocyst-specific genes may not have significant enrichments [55] possibly because of incomplete formation of many organelles (Fig. 1).

**Fig. 2:**
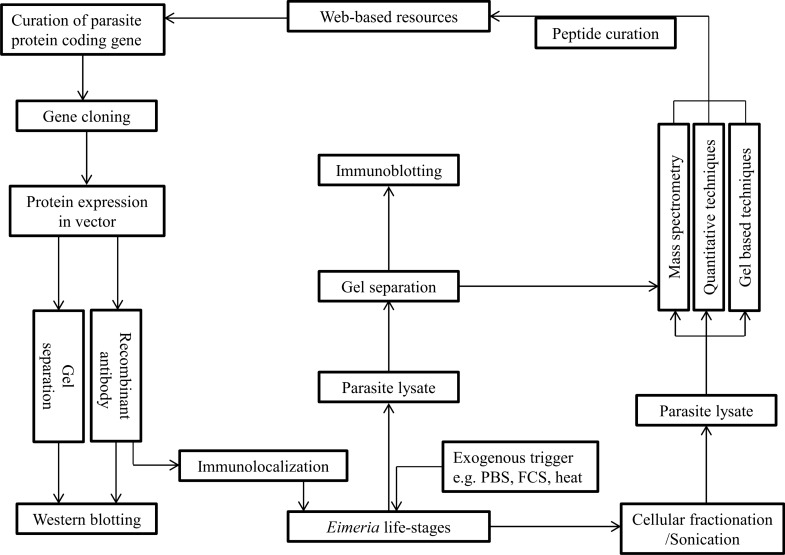
Workflow for protein identification and characterization. Identification and characterization of *Eimeria* proteins are carried out by several biochemical, genetic and in silico approaches. Exogenous stimuli can propel parasites to secrete proteins in vitro and subjection of parasite stages to sonication/organellar fractionation can produce parasite lysates. The crude protein components of parasite lysate can be resolved by chromatography (LC/GC) coupled with gel-based techniques (e.g. SDS-PAGE, 2D-PAGE, 2D-DIGE) and then subjected to MS or MS/MS. Commonly used ionization methods in conjunction with MS include MALDI, SELDI and ESI followed by curation of peptide sequences in the database. Besides, parasite lysate can be subjected to quantitative proteomics techniques (e.g. iTRAQ, ICAT, TMT, SILAC) to identify the relative quantity of each characterised protein curated from web-based library screening. Alternatively, specific protein coding genes could be identified, cloned and expressed in bacterial vectors. The recombinant protein is then used to raise antibody in animals with which protein size (from western blotting) and sub-cellular location (by immunolocalisation/immunofluorescence) of protein in parasite stages are determined. Overall, quantitative proteomics techniques give precise, differential expression of proteins and can predict the underlying functional mechanism that may resolve various overlapping functions of several eimerian proteins. It is however notable that very few studies have used quantitative proteomics methods to characterise *Eimeria* proteins. SDS-PAGE: sodium dodecyl sulphate polyacrylamide gel electrophoresis; 2-DE: two-dimensional gel electrophoresis; 2D-DIGE: two-dimensional differential gel electrophoresis; LC: liquid chromatography; GC: gas chromatography; MS/MS: tandem mass spectrometry; SELDI: surface-enhanced laser desorption/ionization; ESI: electrospray ionization; MALDI-TOF: matrix-assisted laser desorption ionisation time of flight; iTRAQ: isobaric tags for relative and absolute quantification; SILAC: stable isotope labelling by amino acids in cell culture; TMT: tandem mass tag; ICAT: isotope-coded affinity tags

Moreover, *Eimeria* surface antigen genes, *sags*, are of three subfamilies. While *sag*A is widespread in all species, *sag*B is circumscribed to *E. necatrix* and *E. tenella,* and *sag*C is most stretched in *E. brunetti* and *E. mitis* [46]. The number of *sags* is enormous and varies greatly among prominent species [46, 55]. Genome annotation has revealed that pathogenic eimerian can have up to 105 *sags* and species with severe pathologies may have a higher number [46]. Over 80 *sags* have been identified to constitute about 1% of *E. tenella* proteome [59]. However, heterogeneity or nucleotide diversity of protein genes could vary in different isolates [60]. Other prominent protein genes that have been mapped are *hsp90* [57], *hsp70* [61] as well as protease genes in which > 40 are already identified in *E. tenella* genome [62]. Although eimerian developmental stages share many transcriptional and translational products [63], each stage possibly has varying threshold of gene expression [64] and translational profiles [55]. Despite this, eimerian structural and secretory proteins have continued to be characterised and identified by various genetic and biochemical methods (Fig. 2).

## Oocyst and gametocyte proteins

*Eimeria* oocysts can persist in the environment for a long time but they are only infectious when sporulated [32]. Freshly released oocysts become sporulated after exposure to adequate moisture, air and warmth, and the duration of sporulation varies with species (Fig. 1). The structural composition of *Eimeria* oocyst is predominantly scaffolds of protein [65] formed via assemblage of precursor proteins, cross-linking enzymes and cofactors incorporated into wall-forming bodies (WFBs) [66]. On the outer surface of maturing oocysts are the veil-forming bodies, which are electron dense in *E. maxima* [65]. The sequential release of WFB 1 and 2 contents culminated in the formation of eimerian oocyst wall [67]. This is in contrast to *T. gondii* and *C. parvum* oocyst walls that contain carbohydrates and lipids as important structural components [68]. Although eimerian WFB1 are likely to contain glycol- and muco-proteins, the contents of WFB 1&2 are rich in tyrosine [65] and *E. tenella* WFB2 provides essential components for impermeability of the oocyst walls [69]. Additionally, tyrosine motif-containing proteins are prominent among *Eimeria* [50] and the size of WFBs is species-specific with varying antigenicity across coccidian family [65]. Other physiological functions of WFB include gametocyte differentiation and as an integral part of the oocyst wall (Table 1).

**Table 1:** Expression and function of *Eimeria* proteins

Speceis	Stages/organelles	Protein	Functions	References
*E. nieschulzi*	Oocyst	OWP13; cystein-rich	Transglutaminase activity*	[50]
			Oocyst wall formation	
*E. acevulina*	Oocyst, sporozoite	Apartyl proteinase; 43 kDa	Antigen	[153]
*E. tenalla*	UO	LDH, enolase, b-tubulin, kinase Hsp70	Immunogenes, metabolism	[134]
*E. tenella*	Sporoblast, sporocyst SO, sporozoite	Hsp70, 70 kDa	Stress adaptation	[136]
*E. tenella*	Oocyst	Hsp70	SC component, chromosome, pairing, disjuction and recombination	[141]
*E. brunetti*	Oocysts, Sz	MIC2	Immunogenic	[157]
*E. tenella*	SO, Sz, Mz, Tz, schizonts	Protein disulphide isomerase (PDI)	Sporulation, adhesion, invasion and development	[70]
*E. tenella*	SO, Sz, Mz	ESP	Rhoptry, PVM, oocyst microgamete development	[75]
*E. necatrix*	UO, SO, sporocyst wall	gam 22	Immunogene	[53]
*E. tenella*	Sporocyst, SO	MOP1 (28.7 kDa)	Unknown	[72]
	Gametocyte^p^, Sz	MOP2 (30.1 kDa		
*E. stiedai*	UO, SO, Mz	MIC 1 (25.89 kDa)	Immunogenic	[89]
	Gametocyte	MIC 3 (32.39 kDa)	Antigen	
*E. tenella*	OU, SO,Sz, Mz, Tz schizont	ECP (25.4 kDa)	Invasion, development merogony	[110]
*E. tenella*	UO, SO	AMA1	Invasion and development	[94]
*E. tenella*	2nd merozoites, Sz Sz. Mz, UO, SO	SZ-1; 19 kDa, profiling-like	Parasite maintenance	[63]
*E. nieschulzi*	Sporoblast/sporocyst SO, circumplasm	OWP2,6	Sporocyst wall formation	[26]
*E. tenella*	Gametocyte, Mz	Cathepsin-L-like peptidase	Endogenous parasite development, immunogen^p^	[118]
	Sz and UO			
			Initiate sporulation^P^	
*E. tenella*	Sz, Mz, early oocyst, late oocysts	SAGs	Antigen	[71]
		RONs	Protein synthesis, antigen	
		MIC8	Metabolism	
*E. tenella*	Mz	RON2, AMA2& RON5 AMA1& RON4	Cell communication, invasion, antigen	[71]
*E. maxima*	Oocyst	wp29 and wp33	Oocyst wall formation oocyst wall hardening	[66]
*E. maxima*	SO, Sz, Mz	ESP; 30 kDa	Interaction with host structural PVM and microgamete protein	[75]
*E. tenella*	UO, SO, Sz, Mz	ECP 25.4 kDa	PVM formation	[110]
*E. maxima*	UO, SO, Sz, Mz		Antigen	[112, 121, 122]
*E. acevulina*	UO, SO, Sz, Mz			
*E. acevulina*	SO, Sz	Eimepsin/aspartyl proteinase 43-kDa		
*E. maxima*	SO, Sz			
*E. falciformis*	SO, Sz			
*E. tenella*	SO, Sz			
*E. tenella*	Sporocysts, Sz, Mz	ESP (27.9- > 34 kDa)	Sporozoite invasion	[92]
*E.acevulina*	Sz Mz	MIC 3 (93.04 kDa)	Immunogene	[90]
*E. tenella*	Sporocyst	Sporocyst wall protein 1 Tyrosine-rich	Sporocyst wall formation	[158]
*E. acevulina*	Sz, Mz	MIC 5 (12.18 kDa)	Antigenic	[96]
	Sz, Mz	MIC 2	Immunogenic,	[41]
*E. tenella*	Sz, Mz, 1st schizogony	Hsp90	Host cell invasion, stress intracellular growth^p^	[139]
*E. tenella*	Sz	SO7* SAG 13, 14	Invasion antigenic*	[112]
*E. tenella*	Sz, im/mature schizonts	CHP559	Invasion	[33]
*E. acevulina*	Sz conoid	*EF-1α-associated protein	Cytoskeleton, growth, motility, protein turnover, signal transduction, transhydrogenase	[42]
*E. tenella*	Sz apicoplast	38 kDa Malonyl-CoA acyl-carrier protein transacylase	Fatty acid biosynthesis enzyme, drug target*	[150]
*E. tenella*	Sz, 2ndgeneration Mz	Hsp20.4	Sporulation, survival response to stress	[138]
*E. tenella*	Sz	ROP1 (73 kDa) with NTE	Inhibit apoptosis, arrest of G0/G1 cell cycle	[54]
*E. tenella*	Sz, Tz, schizont	MIC2 (50 kDa)	Unknown	[99]
*E. bovis*	Sz, Mz	hsp70-like antigens	Antigenic, parasite survival*	[137]
*E. mitis*	Sz	MIC3; 124 kDa	Antigenic, confers immunity	[98]
*E. tenella*	Sz	MIC 3	Development, invasion	[112]
*E. stiedai*	Sz	100 kDa antigen	Host cell penetration	[109]
*E. maxima*	Sz	IMP1	Immunogenenic	[150]
*E. acevulina*	Sz	p160/p240;19 kDa	(Conserved) antigen	[142]
*E. tenella*	Sz		(Conserved) antigen	
*E. maxima*	Sz		(Conserved) antigen	
*E. falciformis*	Sz		(Conserved) antigen	
*E. tenella*	2nd gen. Mz	14-3-3, subtilase lactacte	Immunogenic	[99]
*E. tenella*	Gametocytes, inner oocyst wall	WFB 2, Gam 22	Oocyst structural component	[57]
*E. maxima*	Gametocytes	Gam 52 and 86	Oocyst wall formation	[64]
*E. tenella*	Sz, Mz and SO	MIC8;100 kDa	Invasion, adhesin immunogene	[85]
*E. acevulina*				
*E. maxima*	WFB, macrogametocytes	gam56, 82 (52.45 and 62.45 kDa)	Antigenic, gametocyte differentiation	[73]
*E. tenella*	Sporozoite	EF-2, 14-3-3, transhydrogenase	Common immunogenic antigens	[108]
*E. acevulina*				
*E. maxima*				
*E. tenella*	Sz, Mz, PVM, immature schizont	19 kDa,175aa;serine/threonine protein phosphatase	Drug resistance, invasion*	[84]
*E. tenella*	Sz, schizont, PVM	MIC1; Transmembrane with epitope 1 &CTR	PV formation, parasite development	[85]
*E. tenella*	Sz, Mz, apicoplast macrogametocyte	35 kDa Enoyl reductase	Type II fatty acid biosynthesis	[40]
*E. tenella*	Sporozoite	Enoyl reductase	Drug target*	[151]
*E. tenella*	UO, SO, Sz	Serpin (45 kDa) serine protease inhibitor	Parasite survival invasion*	[118]
*E. acevulina*	Oocyst*,*Sz, Mz	Serpin; 48/55 kDa	Invasion development*	[94]
*E. tenella*	Sz, 1st & 2nd generation Mz, gametocyte, UO	MIC2;35.07 kDa	Invasion antigen	[58]
		342aa acidic protein		
*E. tenella*	Gametocyte, UO, SO	HOWP1; 40, 30 & 23 kDa	Vaccine*	[26]

*SC* synaptonema complex, *UO* unsporulated oocyst, *SO* sporulated oocyst, *Sz* sporozoite, *Mz* merozoite, *Tz* trophozoite, *speculative protein/function, *NTE* N-terminal extension, *Ef-2* elongation factor 2

Congruently, immunohistochemical analysis indicated similar distribution of WFBs in avian-infecting species with peroxidase and transglutaminase activities of WFB 1 in the formation of isopeptide bonds in oocyst wall [50]. Similarly, protein disulphide isomerase and ally, which catalyse physiological oxidation, reduction and isomerisation of protein disulphide bonds, are mostly expressed in sporulated oocysts of *E. tenella* [70]. Protein disulphide isomerase expression is developmentally regulated and enhances the survival of *Eimeria* and protection from environmentally induced oxidative stress [70].

*Eimeria nieschulzi* outer oocyst wall protein (OWP) 13 is confined to WFB 1 as an orthologous protein in many *Eimeria* species and *T. gondii* with a similar mechanism of cross-linkages via cysteine motif and isopeptide bonding during oocyst wall formation [50]. Conserved *C. parvum* OWP cysteine residues are known to assume disulphide bridges supposedly responsible for stabilisation and formation of oocyst wall. *Eimeria nieschulzi* OWP2 and OWP6 have shown similar amino acid conservation in *Eimeria* and *T. gondii* [26] possibly because of common survival mechanisms outside hosts. Nevertheless, *Eimeria* gametocyte cysteine-rich oocyst wall proteins, orthologues of *Eimeria* cysteine motif containing OWP6, are structural proteins with likely diverse functions in host specificity, oocyst morphology and wall formation, and sensitivity in *Eimeria*, *T. gondii* and *C. parvum* [67]. In general, OWPs are structural building blocks that undergird oocyst wall layers and gametocyte development [49, 65] (Table 1).

Other eimerian OWPs include wp33 and wp29 of *E. maxima* [66] and major oocyst protein (MOP) of *E. tenella* unsporulated oocyst found on the outer portion of sporocysts prior to excystment [49]. MOPs are found in many developmental stages possibly because of alternative gene splicing [50] or catalytic cleavage by subtilisin to form oocyst wall precursor proteins [62] (Table 1). More importantly, sporulated oocysts and late oocysts of *E. tenella* have expressed microneme and rhoptry proteins while unsporulated oocysts (Fig. 1) have shown high superoxide dismutase activity [71]. Identification of microneme and rhoptry proteins in sporulated oocysts is likely because mature sporozoites are already formed and superoxide dismutase activity may include active utilisation of oxygen for sporulation (Fig. 1).

Gametocyte proteins such as gam56 and gam82 have been shown to be involved in the process of oocyst formation in *E. maxima*, *E. tenella* and *E. acervulina* [51], oocyst wall biosynthesis protein (in gametocyte and zygotes) and proteolytic cleavage of OWPs [67]. Again, gam56 and gam82 of *E. maxima* and *E. necatrix* have similar regulatory function [67, 72]. Among *E. maxima, E. tenella* and *E. acervulina*, there are considerable shared characteristics of the gametocyte proteins. However, most notable differences occur in the protein variable sizes [51], which may in turn account for the solubility of gam56 and gam82 antigens [50] but the implication for the oocyst biosynthesis (Fig. 1) is largely unknown. Nonetheless, high molecular weight of gam 56 and 82 might be due to unusual amino acid composition such as high proline content or glycosylation [73].

Coccidian macrogametes are inherently rich in lipids, polysaccharides and precursors of OWP whereas microgametes contain abundant proteins linked to spermiogenesis and DNA condensation [67]. Nonetheless, the formation of oocysts (Fig. 1) results from deposition of gams 56, 82 and 230 from WFBs [71]. It is unsurprising, therefore, that gam56 and gam82 have been detected in early and late oocysts (Fig. 1) but not in the zoite stages [65, 69]. On the whole, gametocyte and oocyst proteins are enriched in tyrosine; in particular, di-tyrosine hydrolysates of *E. maxima* oocysts likely supported tyrosine oxidation during the formation of oocyst wall [66]. It is unknown whether the dityrosine bond in *Eimeria* OWPs is solely responsible for the robust resistant structure of the oocyst. So far, the abundance and localisation of several tyrosine-rich proteins in *T. gondii* oocysts have also given some information to support the possibility that tyrosine linkage maintains the resistance of coccidian oocysts against environmental degradation [54]. Additionally, the oocyst walls of *T. gondii* and *C. parvum* contain cystein- and histone-rich OWPs as important structural components [68] whereas *E. maxima* OWP13 could mediate co-sedimentation or binding of other proteins during oocyst formation (Table 1). The structural protein composition and function during coccidian oocyst wall formation have been adequately reviewed [65] and OWPs, polyketide synthases and transferase enzymes are characteristic of coccidian oocysts [68] but *E. tenella* polyketide synthase biosynthesis pathway has not been functionally determined [74].

## Apical complex proteins

**Fig. 3:**
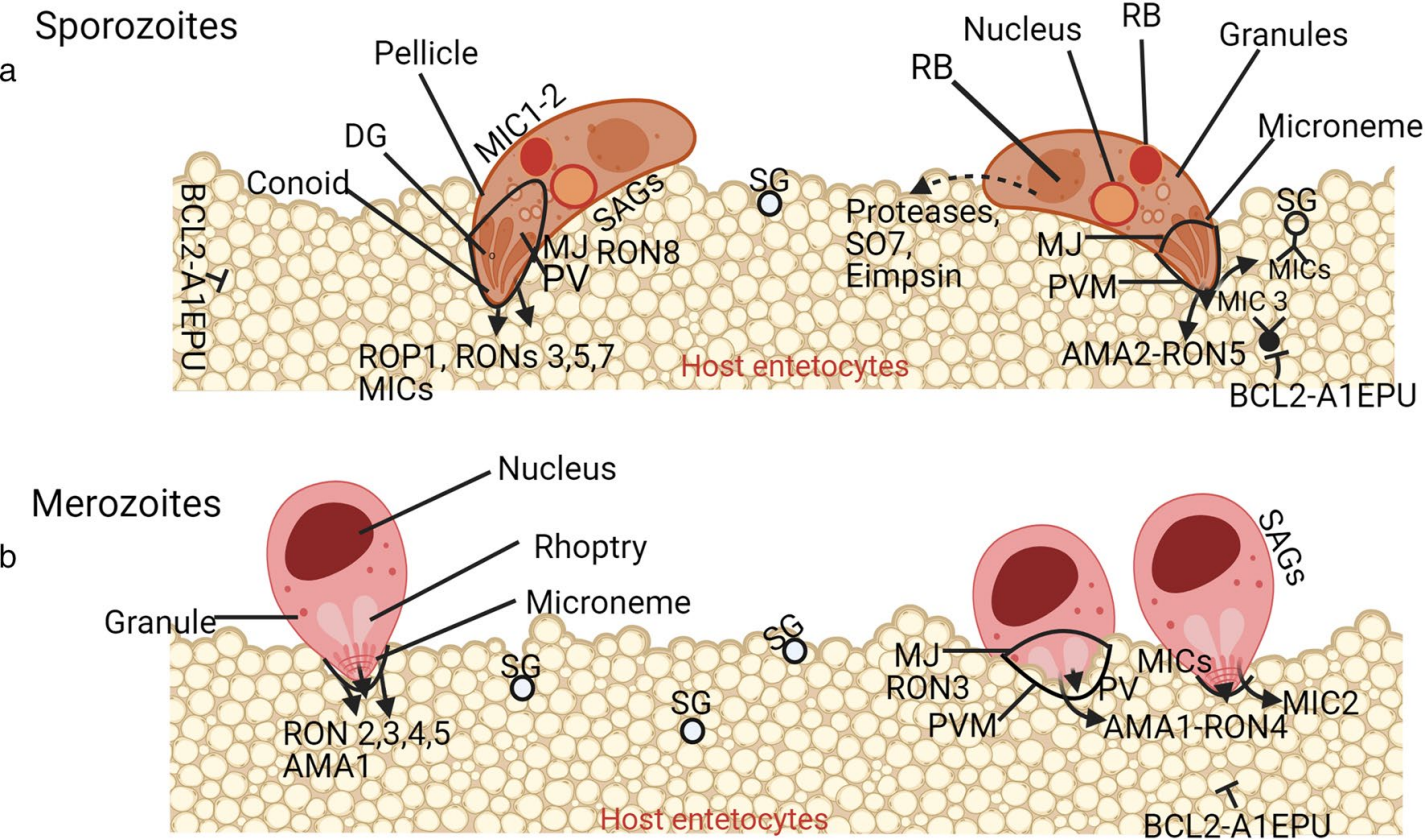
Protein secretion during invasion by eimerian sporozoite and merozoite. **a** Sporozoites must navigate the gut lumen until they reach the enterocytic niche with specific receptor(s) such as BCL2-AIEPU for attachment and which in turn stimulate protein secretion and host SG, which are known to function in the secretion of MICs. At the site of invasion, sporozoites first attach to the enterocytes with a sequence of events including formation of MJ and PVM accompanied by protein secretion. AMA2/RON5 contribute to MJ formation as RBs add to the array of acidic protein secretions. **b** For merozoites, RON4/AMA1 are particularly involved in MJ formation and MICs, ROPs ans proteinase play important roles during the process. Nonetheless, the complexes (AMA-1 and RON4) and (AMA-2 and RON5) may suffice for distinction between swift short-lived merozoites and long-ranging sporozoites. Unlike sporozoites with considerable motility, merozoites invade enterocytes fiercely and locally. The proteins involved during *Eimeria* invasion are quite different from other Apicomplexa [156] probably because of different host cell receptors and *Eimeria*'s extensive migration in host gut. BCL2-AIEPU: associated athanogene 1 and endonuclease polyU-specific-like receptors; SG: surface glycan

Among apicomplexans, rhoptries, micronemes and dense granules are three distinct, unique organelles that comprise the apical complex of zoites [75]. Each rhoptry is club-shaped and secrets two distinct classes of protein, which are rhoptry neck proteins (RONs) and rhoptry proteins (ROPs) secreted from the rhoptry anterior neck region and rhoptry posterior compartment, respectively [56, 76] (Fig. 3). Several ROPs are antigenic epitopes [77] released into parasitophorous vacuoles (PVs) where they modify the vacuolar environment and act as key virulence factors [56]. Formation and function of parasitophorous vacuoles have been extensively reviewed among coccidian genera [5]. Yet, ROPs are divergent across *Plasmodium*, *Toxoplasma* and *Eimeria* [78] and are principally acidic clusters of proteins of around 55 to 65 kDa in *E. tenella* [77] and virulence factor of *N. caninum* tachyzoite [79]. Essentially, *E. tenella* ROP 1 is a kinase protein with catalytic activity that it is conserved among avian eimerian species [54]. Usually, eimerian ROPs are commonly identified after sporulation [71] playing an important role in invasive stages (Table 1) as well as modification of the vacuolar environment, remodelling host cell membrane and protecting the parasite against host defences [56].

In contrast to ROPs, there are about eight RONs with differential expression in sporozoites and merozoites of *Eimeria* and other coccidians [56]. Proteomic analyses have revealed *E. tenella* merozoite RON3, 5, 7 and sporozoite RON2, 3, 4 [71] with more paralogues of RONs (1, 4, 6, 9 and10) in *E. tenella* trophozoites [56]. In a study, RON 2 and 5 have been identified in *E. tenella* sporozoites and comparison of four *E. tenella* life cycle stages indicated differential expression of *E. tenella* RONs [71]. Incidentally, RON 5 and 8 are implicated in moving junction (MJ) (Fig. 3). While RON5 is conserved in *Plasmodium*, RON8 is restricted to *N. caninum*, *T. gondii* and *E. tenella* [80], which thus indicates some degree of evolutionary relatedness. However, the function of individual *Eimeria* RONs within MJ and the presence of additional parasite proteins remain unknown except for RON3, which may perform some roles in invasive stages of *Eimeria* (Fig. 3). Nonetheless, RONs are important in protein synthesis and cell communication (Table 1).

Again, within eimerian zoite apical regions, micronemes are the smallest organelles, which secrete a collection of adhesion proteins, termed microneme proteins (MICs) [81]. MICs are found during development from sporulated oocyst to merozoite stage [71] (Table 1) but are more abundant in sporozoites and merozoites of *Eimeria* than in other apicomplexan genera because of impetuous invasion of enterocytes and migration through intestinal content [7]. Secretion of *E. tenella* sporozoite MICs can be induced through parasite-host cell interaction, in vitro foetal calf serum (FCS) and phosphate buffer saline (PBS) [82] as well as significant temperature change [83] (Fig. 2). Also, heat, cold, chemical factors and nutrition may cause changes in MIC expression [84]. After secretion, MICs persistently appear on parasite membrane and host cell surfaces [85, 86] to enable *Eimeria* sporozoites to bind a diverse range of host cell glycan epitopes [87]. Functionally, MICs are critical for cellular processes including gliding motility, active cell invasion, migration [88] and parasite adhesion [89] (Table 1). Specifically, *E. tenella* MIC2 plays a crucial role in host cell identification and binding [60] just as MIC8 is a key protein in *E. tenella* metabolic processes [71].

By extension, MIC 1–5, 7–9 and apical membrane antigen (AMA) 1–2 have been identified in sporozoites of *E. tenella* [41, 71, 85, 90, 91] with several MIC and AMA orthologues [92]. Similarly, MIC 1, 2, 4, 5 and 7 and AMA 2 have been identified in *E. tenella* second-generation merozoites [93]. *Eimeria* AMA1s have greater homology with those of *Toxoplasma* and *Neospora* than *Plasmodium* and *Babesia* [94] and, again, may possibly be a reflection of phylogenetic similarity among apicomplexans. Like MICs, *E. tenella* sporozoite AMA1 secretion is temperature dependent and its interaction with *Eimeria*-specific protein (ESP) may play a role in parasite invasion, formation of MJ, spliceosomes and immune signalling [95]. *Eimeria tenella* MIC 1 has two epitopes within I and CTR domains. While epitope CTR is relatively conserved, epitope I showed good immunogenicity and varies among species infecting chickens [91].

Although MICs are secreted by similar organelles, they are typically different among apicomplexan genera and species. The amino acid sequence of MIC5 indicated higher homology among *Eimeria* species than in other apicomplexans, but unlike *E. tenella* MIC 1 and 5, *E. acervulina* MIC 5 and *E. tenella* MIC 2 have no trans-membrane signal region for the glycophospholpid anchor [82, 96]; in addition, *E. tenella* MIC2 is soluble with surface capping over the parasite in an actin-dependent manner [82]. Also, *E. acervulina* MIC3 has considerable identity with that of *N. caninum*, *B. bovis*, *P. cynomolgi* and *T. gondii* but somewhat less considerable with *E. maxima, E. brunetti* and *E. tenella* [90, 97]. This indicates that not all MICs are important for host invasions and attachment or homologous MICs may have different functions depending on species (Fig. 3). For instance, *E. tenella* MIC2 secretion is independent of parasite ability to move or invade host cells [82]. There is thus the possibility that the basic function of MICs includes parasite adhesion and formation of glideosome proteins which drive motility [71] and as antigens [98, 99] (Table 1).

*Eimeria mitis, E. acervulina* and *E. tenella* MIC 2 and 3 are concentrated at the apical tip of the sporozoite (but diffused in merozoite) [41, 90, 98], thus suggesting the involvement of some MICs in parasite invasion. This observation is substantiated by *E. tenella* sporozoite MIC3, which has been shown to be a tissue-specific molecule for attachment to the caecal cells via specific ligand interaction with BCL2-associated athanogene 1 and endonuclease polyU-specific-like receptors [100] (Fig. 3). It has been suggested that *E. tenella* MIC1/2 complex is mobilised to the parasite surface during cell attachment and further to the posterior end of the parasite during penetration of the host cell [41, 82]. However, it is unclear why MICs diffused at both poles knowing that sporozoites and merozoites actively penetrate host cells from the anterior apical tip where the microneme is localised. Perhaps, the process of parasite invasion orchestrates re-distribution of specific proteins but this assumption requires further proof. Again, RON/AMA1 complex may be sufficient for host cell entry [80] but the essence and specificity of distinct proteins in the MJ of *E. tenella* merozoite (AMA-1 and RON4) and sporozoite (AMA-2 and RON5) [71] need to be determined (Fig. 3).

After an eimerian has successfully attached to the host cell, the major microneme adhesive repeat region (MARR) proteins are deployed at the parasite-host interface in the early stage of invasion as depicted by *E. tenella* MIC3 [101]. *Eimeria tenella* genome contains MIC 3 with seven Type-1 microneme adhesive repeat (MAR) binding specific spectra of sialyl glycans but from functional analysis, MIC 2, 3, 4 and 5 contain type 1, 3, 4 and 2 MAR respectively. Similarly, *T. gondii* MIC13 has three MAR domains known to bind sialylated glycoconjugates on the host cell [102]. However, MAR sub-cellular location, stage-specific expression and function are yet to be clarified [87]. Interestingly, sialic-acid binding MARRs and carbohydrate-binding domain on *E. acervulina* MIC 3 have been identified [90]. *Eimeria tenella* Type-1 MAR domain containing proteins appears to be expressed within the microneme of *E. tenella* sporozoites invading Madin-Darby bovine kidney (MDBK) cells but its ability to bind a wide range of host cell surface sialic acids and terminal linkages requires more detail [87]. More so, the binding domains of other *Eimeria* MICs are yet to be deciphered. Similar to MARRs, thrombospondin-related anonymous protein (TRAP) family is important for invasion of *Eimeria*. Two typical TRAP proteins, *E. tenella* MIC 1 and 4, have been identified with which *E. tenella* rhomboid protein 3 (ROM3) interacted and may be involved in the cleavage of *E. tenella* MIC4 [103].

Another prominent organelle of eimerian apical complex is dense granules (DGs). DG proteins have been identified in merozoite and during asexual and sexual development of *T. gondii* [38]. DGs are fewer in *Eimeria* compare to *Toxoplasma* and *Neospora* from which about 20 DG proteins have reportedly been found to considerably remodel PVs for parasites intracellular survival [104]. For *T. gondii*, the combinatory complexes of DG proteins and ROPs are integral actors during parasite interaction and invasion of the host cell [105]. However, there has been scanty information on *Eimeria* DGs [106] perhaps because DG genes in *Eimeria* species are few [55] and ROP kinase may function in its stead [54]. Even with the latter assumption, only eimerian ROP1 has been functionally determined (Table 1). Nonetheless, proteins involved in parasite invasion as a component of conoids have long been shown to be conserved in avian *Eimeria* sporozoites and tachyzoites of *T. gondii* and *N. caninum* [107]. Other eimerian apical proteins include TA4, LPMC-61, rhomboid proteins of *E. tenella* and many immunodominant antigens [108] (Table 1).

## Proteins associated with the eimerian apical complexes

Apart from protein secretion from *Eimeria* apical organelles, there have been protein secretions in connection with apical protein repositories. Prominently, pl00 antigen is a major component of micronemes of *E. tenella*, *E. maxima* and *E. acervulina. Eimeria tenella* pl00 antigen is similar to thrombospondin-like protein with two adhesive domains as docks for host cell substance [81]. This protein has a domain that is conserved for antigenic roles in cell-cell or parasite adhesion and may well serve as an analogous parasite receptor [81]. Similarly, *E. stiedai* sporozoite trail antigen is likely to be associated with microneme, with similar immune-reactions comparable to *E. tenella* p100, and may play an important role in parasite attachment and penetration of host cells [109].

In addition, *Eimeria* Specific Protein (ESP) is a protein unique to *E. maxima, E. tenella* and *E. acervulina* with expressed homologous sequences [75] localised to the rhoptry and PV membrane (PVM) around developing oocysts and microgametes [75]. However, ESP is a non-micronemal protein expressed on the surface of permeabilised sporozoites, sporocysts and second-generation merozoites of *E. tenella* (Table 1). Using glutathione S-transferase fusion protein pull-down and bimolecular fluorescence complementation assays, ESP was shown to directly interact with AMA1 of *E. tenella* to mediate sporozoite invasion [92] but the regulatory, phenotypic and genetic consequences of AMA1/ESP complex were not completely elucidated as authors only suggested post-translational modification of these proteins. Similarly, *Eimeria*-conserved protein (ECP) is specific to *E. maxima*, *E. acervulina* and *E. tenella* but its expression is most prominent in sporozoites of *E. tenella* [110]. Indirect immunofluorescence analysis of ECP restricted the protein to the posterior and anterior RBs, apical end of sporozoites and PVM [110] suggesting an important function during parasite entry. That said, apical associated secreted proteins from the zoite apices might have originated from the major secretory organelles but possibly through distinct pathways, and complex interactions with MICs and AMAs also lend some credence. This assumption would likely hold until other organelles are identified in the zoite's anterior regions.

## *Eimeria* surface proteins

Consequent to multi-stage life history, eimerians possess diverse surface antigenic proteins (SAGs) known to be abundant in the invasive stages (Table 1). SAGs are membrane-bound proteins held by glycosylphosphatidylinositol (GPI) anchors to the surface of invasive sporozoites and merozoites [46] and the core function of SAGs appears be attachment to host cells prior to parasite invasion. Currently, *Emerian* merozoites have about 47 SAGs whereas only 4 SAGs have been reported in the sporozoite of *E. tenella* [71] (Table 1). *Eimeria tenella* merozoite SAG, SAG 2, 4 and 19 are localised by a phospholipid anchor on the parasite surface membrane with variations in immunogenicity and abundance [59, 111]. Nevertheless, *Eimeria* SAGs have significant homology with conserved surface antigens of *C. cayetanensis* [59].

Although SAGs show divergence between *Eimeria* species and *T. gondii* [2], they are commonly, like MICs, ROPs and DGs, implicated in host-parasite interaction, invasion and infection [46]. Hypothetically, SAGs assist eimerian merozoite avidity with host cell receptors and thus aid rapid invasion of the short-lived zoite [71] whereas SAG 13 and 14 have been reported to be abundant in *E. tenella* sporozoite [112]. *Eimeria tenella* SAG10 was found across all asexual stages but its transcriptomic expression was found downregulated in drug resistance strains [113] possibly because there were not enough recognisable receptors for drug and host immune response. Nonetheless, the co-expression of SAGs on the surface of invasive and asexual stages of *Eimeria* is reminiscent of a plethora of related epitopes, which potentially could enhance invasion of host enterocytes and immune response just as surface proteins of *Plasmodium* merozoites are important for high antibody response [114]. Suffice to say that the functions of surface proteins at the *Eimeria*-host interface are important to elucidate the mechanism of parasite invasion [115] and therapeutic target. By this, identification and characterisation of SAGs from highly pathogenic species could be ideal in the search for cross-species control targets, drug resistance and susceptibility.

*Eimeria maxima* immune-mapped protein 1 (IMP1) is associated with the parasite surface and has single amino acid substitution that could alter its secondary structure leading to absence of cross-protection among *E. maxima* strains [116]. Three *E. maxima* (APU1, Weybridge and Houghton) strains have been shown to have variable amino acid sequences of IMP1 [101]; however, it remains unknown whether lack of cross-protection among the strains is solely due to variable amino acid sequences of IMP1 or other dominant factors responsible for antigenic variation among the strains. Subtle variability in amino acid sequences of highly conserved proteins among *E. tenella, E. acervulina* and *E. maxima* sporozoites [108] could likely avert cross-immunity. However, this could be explored to identify divergent peptide sequences for antigenic epitopes and immune surveillance. Clearly, deciphering common and distinct surface proteins that serve for antigenicity, immune response or parasite survival will be important in the control of pathogenic *Eimeria* species, especially with respect to therapeutic targets.

## Refractile body and proteases

Proteases, peptidases or proteinases are enzymes that catalyse hydrolysis of peptide bonds in all animal species. Proteases are classified based on their catalytic residues or mechanism as aspartyl, cysteine, serine, threonine and metalloproteases [117]. Proteases facilitate invasion of host cells, digestion of host proteins, host cell membrane degradation and evasion of host immune cells [117]. Proteases are also involved in developmental regulation of protozoan parasites, hydrolysis of proteins, nutrient uptake, and many members of cysteine proteases are major virulence factors of apicomplexans [118]. Typically, many proteases that have been so far identified in *Eimeria* are associated with RB. RBs are notable paranuclear, homogeneous, osmiophilic bodies surrounded by amylopectin granules in *Eimeria* [119]. Of all organelles in *Eimeria* sporozoites, RBs show prominence but reduce in size and eventually wane after the first schizogony [120, 121]. The functions of RBs as distinct organelles of Eimeriidae are still being unveiled [33]; however, *E. tenella* RBs have only been found in sporozoites and trophozoites and proteomic analysis has confirmed that RBs are reservoirs for acidic proteins [120] (Table 1).

Aspartyl proteinases from *E. tenella* sporozoite RBs and other stages have been reported [122] (Table1). In effect, RB proteins such as aspartyl proteases, eimepsin and SO7 belong to several protein family members including haloacid dehalogenase, hydrolase, subtilase, lactate dehydrogenase and ubiquitin. Eimepsin is perhaps one of the well-characterised *Eimeria* proteases with four (I-IV) antigenic domains in which domain I, III and IV changed dramatically at the apices of invading sporozoites whereas antigenic domain II is located in RBs [123]. Similarly, SO7 is an immunogen with conserved antigenic epitopes in *Eimeria* species infecting domestic fowl. SO7 has an important role in host cell invasion and secretion of MICs and may also function in parasite intracellular survival [124]. In addition, a transhydrogenase found in *Eimeria* RBs might also function in ATP hydrolysis and respiration during sporulation [108]. Although eimepsins belong to the aspartyl proteinease family, which is largely produced during sporulation [123], in the sequence of development, RBs are only found after sporulation [71] as confirmed by the abundance of eimepsin in *E. tenella* sporozoite [112]. It is thus likely, at least for eimepsin, that protein expressions and formation of reservoir organelles are consequent events but it is unclear whether proteins are stored in active or precursory form.

Of the four major catalytic classes of peptidases, only aspartyl proteases are developmentally regulated in *Eimeria* during oocyst sporulation [125]. Aside from developmental regulation, serine proteases could mediate *Eimeria* sprozoite cellular invasion [126] that is accompanied by shedding surface adhesins by proteolysis mediated by rhomboid protease [103]. Parasite rhomboid proteases are known to enzymatically cleave other proteins and cell surface adhesins.Especially, *E. tenella* ROM3 played important roles in cleaving *E. tenella* MIC4 [127]. Serine proteases related to rhomboid proteases are equally involved in protein processing of micronemes [126]. Also, *E. tenella* proteases were among highly upregulated transcriptional regulators of parasite life cycles, attack tricks and egress from host cells [128]. Unsurprisingly, proteases have been described in all developmental stages of *E. tenella* [129] but not in other pathogenic species (Table 1). However, the function(s) of proteases in non-invasive stages of *Eimeria* have not been fully elucidated.

Gleaning from the biology of *Plasmodium* and *Toxoplasma*, the roles of proteases revolve round invasion, egress, cellular degradation and protein homeostasis [130]. Remarkably, serine protease inhibitors (serpins) are secreted to protect invading parasites from degradation by host-derived proteases. The secretion of *E. tenella* sporozoite serpin has been triggered in vitro by PBS and culture media (Fig. 2) with a homogeneous cytoplasmic distribution pattern that was more concentrated at the parasite apical end [106]. However, a fundamental stimulus that triggers such anti-host serpins in *Eimeria* has not been fully deciphered. Equally, serpins are likely to have species-specific functions because *E. acervulina* serpin did not show inhibitory activity against host serine proteases [106] unlike serpin from *E. tenella* [131] even though both species infect chickens. It is necessary therefore to characterise parasite and host proteases that are targets for *Eimeria* serpins because (1) proteases are substrate specific, (2) protein export/translation may partly change because of proteolysis and (3) parasite-host crosstalk may also involve inter-reactivity of host- and parasite-derived proteases [130]. Such knowledge would expand our understanding of host cell lysis and immune evasion during parasite-host interactions [131]. In addition, typical serine protease inhibitors reduced *E. tenella* sprozoite invasion in vitro and the localisation of serpins in yet unidentified granules may also suggest a secretion via distinct pathway [106].

Exceptionally, the secretion of *E. tenella* cathepsin-L-like peptidase decreased during sporulation [118](Fig. 1). Also, alkaline proteases are present in all developmental stages of *E. tenella* with strong homology to subtilisin and oligo-endopeptidase [129]. In a similar manner, *E. tenella* aminopeptidase (AP) is highly expressed during sporulation but absent or conspicuously reduced in sporozoite and merozoite stages, and variant forms of AP, such as leucine in *E. falcimformis* and *E. tenella* sporulated oocysts, share significant homology with other apicomplexan AP [132] playing important roles during host cell invasion, immune responses, peptide digestion and excystment [126] (Table1).

Sporulation of oocysts in coccidian involves metabolism of large quantities of carbohydrates by enzymes such as GAPDH, lactate dehydrogenase and superoxide dismutase [108, 132]. In *Eimeria* schizont, glycolytic enzymes, such as enolase, possibly support nuclear activity for energy production and anaerobic adaptation of intracellular stages and exystation of sporozoites [133]. Also, enolase and kinase are important *E. tenella* immunogens [134]. Western blot and qPCR analyses have demonstrated that *E. tenella* serine/threonine phosphatase (STP) was highly expressed in drug-resistant compared with drug-sensitive strains. The association of STP with drug resistance may possibly be linked to mutation with contiguous genes encoding proteins that interact with STP [84]. Of the enzymes secreted via *E. tenella* apicoplast, enoyl reductase is important in the formation of fatty acid synthase and synthesis of type 1 and 11 fatty acids [40] but multiple pathways for fatty acid synthase geared toward various organelles [74] need further elucidation.

## Cytoplasmic proteins

Although MIC2 and serpins have been found in the cytoplasm of some eimerian developmental stages [58, 106], heat schock proteins (Hsps) are pervasive cytoplasmic proteins with distinct subsets confined to mitochondria. Generally, Hsps are chaperones for protein precursors, secretions, transport, folding, assembly and biosynthesis [135]. The secretion of Hsps may be constitutive or synthesised in response to heat-induced stress [135] during infection, chemical and mechanical stimulations, and the excystation process [136, 137]. Secreted Hsps mediate equilibrial temperature of parasites in relation to the surrounding and also prevent protein aggregation [138]. Invasion of host cells often enhances secretion of parasite Hsps in response to higher host temperature or stress during barrier breakage [136] and development within the hosts [139]. Essentially, Hsp90 is dispersed within cytoplasmic and pre-nuclear regions of all *E. tenella* life stages and PV but not in micronemes and rhoptries. Nevertheless, Hsp90 is an active protein necessary for invasion and could play a number of roles in signal events for the secretion of MIC and RON complexes and regulation of host-parasite interaction through signal transduction pathways [139].

At least two homologues of Hsp70 have been reported in relation to conservation and ubiquity. These include cytosolic Hsp70 of *E. acervulina* and *E. maxima* [138] and mitochondrial Hsp70 of *E. tenella*, which presumably is synthesised on cytoplasmic ribosomes after which its signal sequence is directed to the mitochondria [135]. In addition to *E. acervulina* Hsp70 [111], antigenicity of three Hsp-like proteins has been reported in *E. bovis* sporozoites and merozoites as cognates of *P. falciparum* merozoite 75-kDa Hsp [137]. A significant gradual decrease in the expression of Hsp70 in sporozoites of wild and precocious strains of *E. tenella* during continuous attenuation has been reported. While Hsp70 cytoplasmic distribution was observed in the entire sporozoite of the wild strain, it was reduced to the anterior portion in the precocious lines [140]. It, however, remains unknown whether abundance of Hsp70 in wild *E. tenella* correlates with virulence.

Despite this, Hsp70 plays an important role in the formation of sporocysts and sporozoites [61] (Table 1). A dose-dependent inhibition of Hsp70 by quercetin inhibited the formation of syneptonema complex and haploidy in *E. tenella* sporozoite suggesting that Hsp70 could act as sentinel for assembly and disassembly of other proteins during developmental transition [141]. Operationally, *E. tenella* Hsp70 is a molecular chaperone critical for the maintenance of cell homeostasis by enhancing immunogenicity elicited by *E. tenella* MIC2 [138, 142]. Also, *E. tenella* Hsp70 and Hsp90 can form multimers or hetero-complexes with other parasite proteins as observed in *E. tenella* sporozoites [139]. However, the importance of the interaction is unknown. Other Hsps include *E. tenella* Hsp20.4, which is a distinct variant of Hsp20 protein family. *E. tenella* Hsp20.4 contains Hsp20/alpha-crystalline domain, which determines its function as molecular chaperone, and it is likely to be involved in sporulation and intracellular development [138].

## Hindsight

Factors inherent in eimerian biology and experimental procedures influence protein identification, expression [139] and conformations [73, 134]. Also, antibody may not recognise parasite extracts ab initio [72] (Fig. 2) because of protein self-activation/re-naturation [118, 143], isoforms and clusters [42, 134]. Various *Eimeria* stages may show simultaneous or differential expression of some proteins [112, 144], which invariably depend on level of expression, importance to parasite stage, host response [91], limitation (or liberality) of fluorescent antibody [66] and gene splicing [52]. In addition, some proteins may be undetected because of inherent difficulty to reproduce in in vivo conditions.

Protein interactions can affect diverse cellular functions [92] but protein size is de facto insufficient and limiting (Table 1) except if converted to a peptide sequence [145]. Meanwhile, obvious challenges with mass spectrometry include decoy search strategy [146], correct peptide identification [91, 112] and intractable genome annotations [144]. Expression of protein can be hindered in situ by lack of correlation between transcription and translation [39, 89, 113]. As well, RNA degradation can cause transcriptional suppression [147] of protein mRNAs [148] and hence obstruct translational events [149]. Protein may be dormant outside its functional site [150] and so identification at this stage may not indicate functionality. There could be conformational differences between natively secreted and cloned proteins [90, 106, 151] and isolation of clones without biological relevance [92] is possible. Also, specific protein from different isolates (precocious and wild type) and strains might differ significantly [152].

## Future outlook

Characterisation of conserved proteins may help to identify potential antigens [153] (Table1). *Eimeria* proteins such as proteases and Hsps from field strains may give significant antigenic clues [8] and help our understanding since precocious strains  can secrete proteins that are variants of ‘precise’ virulence factors in the wild type [112]. Identification of protease-mediated processes would facilitate better understand of host cell lysis and immune evasion [131]. Factors influencing changes in amino acid sequences such as single nucleotide polymorphisms [101], mutation and antigenic variation [84] and trypsinic hydrolysis [112] need to be completely defined. Identifying when and why these changes occur will be essential to explain some mechanisms of antigenic variation, drug resistance and immune subversion.

Development of new therapeutic targets depends on the discovery of parasite gene products [108] but large tracts of protein-coding genes are yet to be functionally analysed [56] and mapped [40, 151]. Application of forward and reverse genetics will provide further insights into the structural simulations and protein compositions. Also, *Eimeria* proteins that are secreted via distinct vesicles [65] and granules [106] need to be appropriately characterised as in other protozoan parasites [154]. In-depth proteomic profiling that includes RNA-Seq, quantitative proteomics and mass spectrometry (Fig. 2) would unveil key antigens and offer cognate clues about immunogenic proteins [23] compared with expression in plasmids. Instead of a single proteomic approach, high throughput and quantitative proteomics techniques are advocated for functional characterisation of *Eimeria* proteins [155].

## Conclusion

Eimerian secretory and structural proteins are important for survival, physiological adaptation, pathogenesis and antigenicity. Moreover, these proteins differ in expressions, compositions and functions depending on parasite species/strains, developmental stages and stimulations from host cell receptors and exogenous triggers. We have only given a conspectus on the current spectrum of *Eimeria* proteins; nevertheless, it is anticipated that future application of new generation proteomics techniques, proteogenomics tools and identification of other eimerian secretory pathways will aid protein characterisation.

## Data Availability

Not applicable.
